# The Castle of Indolence

**Published:** 1921-03-19

**Authors:** James Thomson


### HOSPITALS IN LITERATURE.

### "The Castle of Indolence," by James Thomson.

Thomson was a typical eighteenth-century English poet. He is best known for his long poem called "The Seasons," and (a fact not quite so familiar) he is the author of "Rule Britannia."

"The Castle of Indolence" is a very fine poem, written at the height of his power and fame, consisting of two cantos, Canto i. containing 78 and Canto ii. 79 stanzas.

In the first part, Canto i., is depicted a place of which the ruling idea is that in it everyone who enters may be set free from all that prevents an easy-going, indolent, joyous life, and the attractions of having no labour or toil of any kind are particularly emphasized. This region is called the Castle of Indolence, but includes pleasant meadows, rivers, streams, etc., such as Thomson delighted to versify: and over all presides a beneficent-looking, but really evil-minded, wizard.

The first canto closes down with the dark and dismal results of this deceptive life of indolence, and the wizard appears in his true colours as the enemy of men, working their misery and ruin. And here it is noticeable how the poet works in such unpoetic words as disease, hydropsy, lethargy, spleen, hypochondria, gout, apoplexy, without ruining the poem. Indeed, the line that speaks of gout is one of the most able in the canto—

The sleepless Gout here counts the crowing cocks.

A reference to hospitals is made in the stanza (77) which describes the character of Hypochondria, which is personified:

She felt, or fancied in her fluttering mood,
All the diseases which the *spittles* know.

But we will return later to this form of the word "hospitals." There is no need to trace in full the adventures and exploits of the Knight of Industry, how he rescues, by his eloquent reasoning, from "that soul-enfeebling wizard Indolence" all who are moved to penitence. But body, as well as soul, has been enfeebled, and calls for remedial methods. Consequently we read, after a fresh waving of the Knight's "powerful wand":

(72) When lo! a goodly hospital ascends,
In which they bade each lenient aid be nigh,
That could the sick-bed smooth of that sad company.

(73) It was a worthy edifying sight,
And gives to human kind peculiar grace,
To see kind hands attending day and night,
With tender ministry, from place to place.
Some prop the head; some from the pallid face
Wipe off the faint cold dews weak nature sheds;
Some reach the healing draught; the whilst, to chase
The fear supreme, around their softened beds,
Some holy man by prayer all opening heaven dispreds.

Probably no finer picture of a hospital at work can be found in English poetry.

We will not stop to consider the "lazar-house" of ii. 67, and the "pesthouse" of ii. 68, both forms of hospitals, but let us confine our attention to two points, one literary and etymological, the other historical: (1) The word "spittles"; (2) in the picture of the "goodly hospital," have we any reason to suppose that such institutions existed at the time (A.D. 1748), or must we conclude that Thomson was idealising?

It will be convenient to take (2) first. Inasmuch as the age produces the man, and Thomson was the product of his age, the passage shows that the idea of hospitals worked as they are in our own day was at least in men's minds. But whether such a hospital existed when Thomson wrote or in any subsequent part of the eighteenth century is another question. And if "goodly hospitals" were familiar facts, the poetry of that line is bad; if the poetry is good, the notion is ideal.

Yet the age was truly philanthropic, and in Thomson's period the following important hospitals were founded—St. George's; London; Middlesex—and as to the administration, a weighty authority (Gray "History of English Philanthropy") writes: "The treatment of patients, whether in the matter of nursing or diet, was no doubt rather rudimentary, yet all the evidence goes to show that considerable thought was given to make the condition of the inmates as comfortable as possible." This comfort, by the way, did not extend to a cup of tea, which was strictly forbidden, though beer was allowed!

We now turn to the ugly-looking word "spittles." This form of the word "hospitals" has been reached by a verbal process known as *aphetesis*. It is an "aphetised" word. Many words in popular use come to lose either the first or the last portion. Examples are: Cute for acute; bus for omnibus; paper for newspaper; squire for esquire. So hospital became in speech and writing either spittle or spital. A few instances of this are:

Shee whom the spittle-house would cast the gorge at.—*Shakespeare.*

I ha' nine darters i' the spital.—*Tennyson, Becket.*

Such a nauseous spital.—*Smollett.*

May they lie and starve in some miserable spittle.—*Ben Jonson.*

It will be noticed that in all these cases the word is used contemptuously, leaving us to infer that the hospital of the period was not a very inviting place.

